# Contrasting life‐history strategies of three sympatric icefish species in the northern Scotia Sea

**DOI:** 10.1111/jfb.70344

**Published:** 2026-02-10

**Authors:** Huw W. James, Timothy Jones, Fabrice Stephenson, Philip R. Hollyman, William D. K. Reid, Martin A. Collins

**Affiliations:** ^1^ School of Natural and Environmental Sciences Newcastle University Newcastle upon Tyne UK; ^2^ British Antarctic Survey Natural Environment Research Council Cambridge UK; ^3^ School of Ocean Sciences Bangor University Menai Bridge UK

**Keywords:** Channichthyidae, icefish, life history, Scotia Sea, South Georgia, Southern Ocean

## Abstract

Comprehending a species' life‐history strategies is crucial to inform effective conservation efforts. Commercial fishing impacts icefish (family: Channichthyidae) in the Scotia Sea, but detailed information on species‐specific life histories remains largely unknown. In this study, the demographic characteristics of mackerel icefish (*Champsocephalus gunnari*), blackfin icefish (*Chaenocephalus aceratus*) and South Georgia icefish (*Pseudochaenichthys georgianus*) were examined and used to compare inferred life‐history strategies, using long‐term data from demersal and plankton trawl surveys conducted across the South Georgia and the Shag Rocks continental shelves. The results indicated that *C. gunnari* may exhibit alternative reproductive tactics, as they appear to spawn multiple times in a single year and mature at varying sizes. Conversely, *C. aceratus* and *P. georgianus* reproduced once per annum and appeared to favour investing in somatic growth, resulting in them consistently maturing at a larger size. Seasonal sex ratios demonstrated a reduction in captured mature males for *C. aceratus* and *P. georgianus* during the suspected spawning period, supporting the hypothesis of sex‐specific behavioural patterns during the reproductive period. These findings highlight the variation in the life‐history strategies among these three icefish species, which should be considered during the development of future management measures.

## 1 INTRODUCTION

Demographic characteristics, including the reproductive strategy, age structures and the size that sexual maturity is attained, can provide crucial information on the life‐history strategies of a species (Awruch et al., 2021; Clarke et al., 2008; Winemiller & Rose, 1992). Such traits can help comprehend a species' vulnerabilities to changing anthropogenic and environmental pressures (Isaac, 2009; King & McFarlane, 2003; Musick, 1999). For example, the resilience of species that mature at a comparatively fast rate, reproduce frequently and expend little to no energy into parental care differs from those that mature slowly, have a high energy expenditure on reproduction and reproduce infrequently (Le Bris et al., 2015; Rolland et al., 2020; Sadovy, 2001). Furthermore, life‐history traits can vary between closely related species and even among populations within a single species (Christiansen et al., 2008; Kock, 2005). Due to these factors, identifying a fish species' key life‐history traits across its geographic range is critical for the sustainable management of fisheries resources (Erisman et al., 2017; King & McFarlane, 2003; Parker et al., 2019). However, collecting data on life‐history traits in fish is often challenging, and species‐specific information is frequently lacking, especially for those endemic to the Southern Ocean (Sands et al., 2024; Van De Putte et al., 2021). The scarcity of data makes selecting appropriate management strategies challenging (Erickson & Nadon, 2021; Prince et al., 2023; Willse et al., 2024). This is true for icefish (family: Channichthyidae), whose life‐history information remains fragmented or largely unknown.

Mackerel icefish *Champsocephalus gunnari* Lönnberg 1905, blackfin icefish *Chaenocephalus aceratus* (Lönnberg 1906) and South Georgia icefish *Pseudochaenichthys georgianus* Norman 1937 have been impacted by commercial fishing operations in the Scotia Sea. *C. gunnari* was targeted extensively in multiple sub‐Antarctic regions, with total catch exceeding 200,000 t during the 1983 fishing season (CCAMLR, 2019). *C. aceratus* and *P. georgianus*, listed as Vulnerable and Endangered, respectively, by the IUCN (Williams, 2024a, 2024b), were primarily caught as by‐catch but were occasionally targeted in the 1970s and 1980s when *C. gunnari* catch rates had declined due to overfishing (Kock, 2005). The waters on the continental shelf surrounding the sub‐Antarctic Island of South Georgia are an example of where the populations of each species have been over‐exploited. Towards the end of the 20th century, stocks of the main target species were deemed overfished leading to the closure of the fishery in the early 1990s (CCAMLR, 2021; Fallon et al., 2016). After reopening the mackerel icefish fishery in 1995 (CCAMLR, 2021), more stringent management was introduced. These measures included restricting fishing to pelagic trawls, setting a precautionary annual catch limit for *C. gunnari*, which reached a maximum of 6760 t in 2001 and further targeted fishing for *C. aceratus* and *P. georgianus* was prohibited (Abreu et al., 2024; CCAMLR, 1990, 2021; Fallon et al., 2016; Kock & Everson, 2003; Kock & Köster, 1990; Trathan et al., 2021). Previous studies suggested that the recovery of icefish populations in the Scotia Sea was ongoing towards the end of the 20th century (Kock & Everson, 2003; Kock & Köster, 1990).

Several gaps persist in the current understanding of the life history of *C. gunnari, C aceratus* and *P. georgianus*. These include uncertainties regarding their reproductive traits, such as the timing and frequency of spawning events, energy expenditure for reproduction and the size at maturity. Investigating these traits could provide important information to understand regional population dynamics that will support effective management measures in relation to fisheries and a changing climate. For example, there is a growing body of evidence to suggest that multiple icefish species exhibit parental care, most commonly by building nests and brooding their eggs during the incubation period (Detrich et al., 2005; Ferrando et al., 2014; Kock et al., 2006, 2008; La Mesa et al., 2022; Purser et al., 2022). *C. aceratus* nesting sites have been identified in sub‐Antarctic regions (Detrich et al., 2005), but as yet, none have been found in the northern Scotia Sea. Furthermore, there are still uncertainties regarding the basic reproductive habits of both *C. gunnari* and *P. georgianus*. *C. gunnari* are reported to spawn annually within South Georgia's fjords (Kock, 2005; Kock & Everson, 1997), resulting in the adoption of management measures prohibiting fishing within 12 nautical miles of South Georgia during their suspected spawning period (CCAMLR, 2024). However, there is evidence to suggest that multiple spawning periods may occur in a single year (Belchier & Lawson, 2013; North, 1998). As reproductive strategies are often used to inform fisheries management efforts (CCAMLR, 2022; Erisman et al., 2017), it is vital to build on the current understanding of the life‐history strategies of South Georgia's icefish.

This study aimed to build on work conducted by Clarke et al. (2008) and Reid et al. (2007), by using data sourced from 22 years of plankton surveys (2001–2022) and 26 demersal trawl surveys (1986–2023) conducted across the South Georgia and Shag Rocks continental shelves. The resulting data were used to infer aspects of each icefish species' life‐history strategies, which were then compared. Specifically, we investigated (1) monthly abundance and length frequency of *C. gunnari, C. aceratus* and *P. georgianus* larvae; (2) the size at maturity and seasonal variation in sex ratios; and (3) the length range of cohorts and the proportion of individuals at different maturity stages within those cohorts. The results were used to compare the life‐history strategies that each species exhibits.

## 2 METHODS

### 2.1 Study site

**FIGURE 1:**
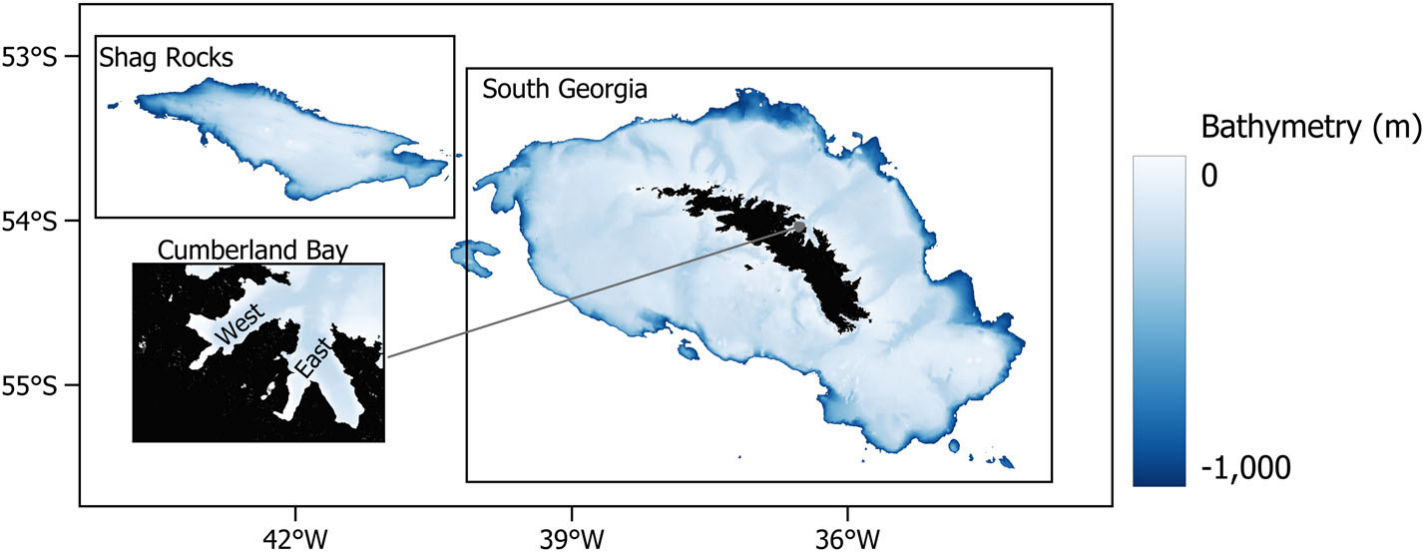
The study sites of the South Georgia and Shag Rocks continental shelves along with an inset map detailing the location of Cumberland Bay. Bathymetry data were curtailed at 1000 m (Hogg et al., 2016, 2017), and the coastline data were sourced from South Georgia GIS (2019). The figure was produced using QGIS 3.34.5‐Prizren (QGIS Development Team, 2024).

The study area extended across the continental shelves of South Georgia (≈53–56° S; 40–34° W) and Shag Rocks (≈53–54° S; 44–40° W) (Figure 1). The shelves encompass an area of 39,750 and 9200 km^2^, respectively, and are separated by approximately 30 km (Allcock et al., 1997; Fretwell et al., 2009; Hogg, 2018; Hogg et al., 2011). Each region lies to the south of the Polar Front and is in the path of the Antarctic Circumpolar Current (Ward et al., 2002). Shag Rocks has lower species richness in its demersal fish community in comparison to South Georgia (Gregory et al., 2017), which possesses one of the highest levels of biodiversity in the Southern Ocean (Hogg et al., 2011).

### 2.2 Plankton survey

Plankton trawls were focused across the inshore regions of South Georgia with the majority conducted in Cumberland Bay (Figure 1). A lower number of trawls were undertaken across the continental shelf of South Georgia, along with four trawls at Shag Rocks. The trawls were completed between 2001 and 2022 (Figures S1–S4), using a rectangular midwater trawl with a 1 m^2^ opening and a 610 μm net mesh. Tows lasted approximately 30 min at a maximum speed of four knots. The sampling was conducted at a depth of 25 m to avoid the freshwater surface layer and at a depth where fish larvae were known to be present (Belchier & Lawson, 2013). A second repeated trawl was made where possible, where the tow was completed in the opposite direction of the first or consecutively in a straight line. Fish larvae were identified to species level, counted and measured for standard length (SL) to the nearest millimetre (Belchier & Lawson, 2013). The swept volume was used to calculate the number of individuals caught per 1000 m^3^ of seawater filtered. The mean monthly catch per unit effort (CPUE) was then plotted along with 95% confidence intervals calculated via the bootstrap method, which were truncated at zero (Figures S1–S3). Ridgeline plots, using the package *ggridges* (Wilke, 2024), were produced to visualise the monthly distribution of larval lengths.

### 2.3 Demersal survey

Data on the abundance, distribution and characteristics of juvenile and adult *C. gunnari, C. aceratus* and *P. georgianus* were collected during 26 demersal trawl surveys conducted between 1986 and 2023 (Table S1). The continental shelves of South Georgia and Shag Rocks were fished during each survey (Figure 1), except for 1989 when the Shag Rocks region was not sampled. Pre‐1990, the demersal survey was undertaken to assess the impact of heavy exploitation of Southern Ocean fish populations. Since 1990, the focus has shifted to providing biomass estimates for *C. gunnari* as well as the distribution and abundance of juvenile Patagonian toothfish *Dissostichus eleginoides* Smitt 1898. The survey design has remained relatively constant over the timeframe, except for changes in the fishing vessel and the number of depth zones, to ensure comparability (Saunders et al., 2022). The survey area was initially divided into three depth strata, 50–149, 150–249 and 250–500 m, which was later reduced to two depth zones; 50–200 and 201–350 m due to the low number of hauls completed in the shallowest strata. The maximum depth generally remained at 350 m, except for deepwater trawls conducted in 2003, 2010 and 2019, which achieved a maximum depth of 964 m. Sampling locations were randomly spread around the study area using a grid, measuring 0.5° latitude by 1 degree of longitude, except for 2003 where trawls were undertaken in a series of non‐random transects radiating outwards from the South Georgia landmass (Collins et al., 2004; Gregory et al., 2017). Trawling occurred across both continental shelves, except for areas where the seabed was unsuitable (Figure S4). All trawls employed a ground bottom trawl method and predominantly an FP‐120 net with a codend of 80 mm and a liner of 40 mm (Belchier et al., 2015), except for surveys conducted between 1986 and 1989 (Table S1). Trawls fished on average for 30 min at approximately 3.7 knots, resulting in a mean trawl distance of 1.8 nautical miles. Twenty‐two surveys were conducted from late November to early February and were grouped as surveys conducted in the Austral summer. In 2008 and 2021, the demersal survey was conducted in April and May (henceforth referred to as Austral autumn surveys), and in 2007 and 1997, the surveys were conducted in August and September (referred to as the Austral winter surveys) (Table S1).

The total catch from each haul was sorted, and all fish were identified to species level. For each fish species, the total catch weight (kg) per trawl was recorded along with the number of individuals caught, which was estimated during large catches. All fish were measured for total length (TL) (to the nearest centimetre below) where possible, with priority given to *C. gunnari* and *D. eleginoides*. For catches above 300 individuals, a random subsample of approximately 200 to 300 individuals was measured, and the sampling factor was recorded. On some occasions, the samples were stratified to ensure the inclusion of larger individuals. For full biological analysis, a maximum of 30 individuals were non‐randomly sampled across the available size range per haul per species. Priority was given to fish over 20 cm in TL because of the challenge of sexing and maturing individuals below this size. Full biological analysis included recording the total weight (g) using motion‐compensated scales, and macroscopic examination of their gonads to assess sex and maturity stage. Maturity was assessed against the five‐point scale developed by Kock and Kellermann (1991): maturity stage 1 = Immature; maturity stage 2 = Resting/Developing; maturity stage 3 = Developed; maturity stage 4 = Gravid/Ripe; and maturity stage 5 = Spent.

### 2.4 Maturity and length at 50% spawning analysis

Maturity within the populations of the three icefish species was examined by plotting the proportion of maturity stages per centimetre size interval. The size at first spawning (stages 3–5) was calculated by analysing the relationship between the cumulative proportion of Stage 3 and above and total length by fitting a logistic regression (Kock & Kellermann, 1991). This allowed for the calculation of length at 50% spawning (L_50_) for each sex per species (Hollyman et al., 2021; Kock & Kellermann, 1991), with confidence intervals derived from bootstrap resampling, using the *FSA* and *car* packages (Fox & Weisberg, 2019; Ogle, 2018; Ogle et al., 2025; Pope et al., 2010). A bootstrap hypothesis test, using 10,000 iterations, was conducted to compare the L_50_ values between the sexes. Fish below 20 cm (TL) were assumed to be immature due to the difficulty in sexing and maturity staging small individuals. The sex ratios for both mature and immature individuals were plotted for each species for the Austral summer, autumn and winter. All analysis was completed using R version 4.5.0 (R Core Team, 2025), and the figures were produced with a combination of the packages *ggplot2* (Wickham, 2016), *ggpubr* (Kassambara, 2023) and QGIS 3.34.5‐Prizren (QGIS Development Team, 2024). As previous research has suggested that the populations of *C. gunnari* at South Georgia and Shag Rocks could be separate stocks (Kuhn & Gaffney, 2006), fish attributed to each region were analysed separately.

### 2.5 Putative age cohort identification

Length frequency data were used to identify size cohorts that represented homogeneous age groups for each species. To guide the identification process, the *mixR* package (Yu, 2022) was used to fit finite mixture models (FMM) to the length frequency data for each survey year and species. Length cohorts were then identified for each season that surveys were completed. A Gaussian distribution and unequal variance were assumed for each cohort. Models were trialled with varying numbers of cohorts per species (*C. gunnari* = 2–5 cohorts for both South Georgia and Shag Rocks, *C. aceratus* = 4–8 and *P. georgianus* = 2–5). FMMs fitted with different numbers of cohorts were compared using the Bayesian information criterion (BIC), and the model with lowest BIC was selected to represent the number of cohorts present for each season and species. The FMMs were then used to estimate the length range of each cohort. In some instances, the FMM would identify a pseudo‐cohort or would suggest splitting a cohort that was likely to represent a homogeneous age group. In these instances, the length range of the cohorts was adjusted to those that were most ecologically relevant.

## 3 RESULTS

### 3.1 Larval temporal abundance

**FIGURE 2:**
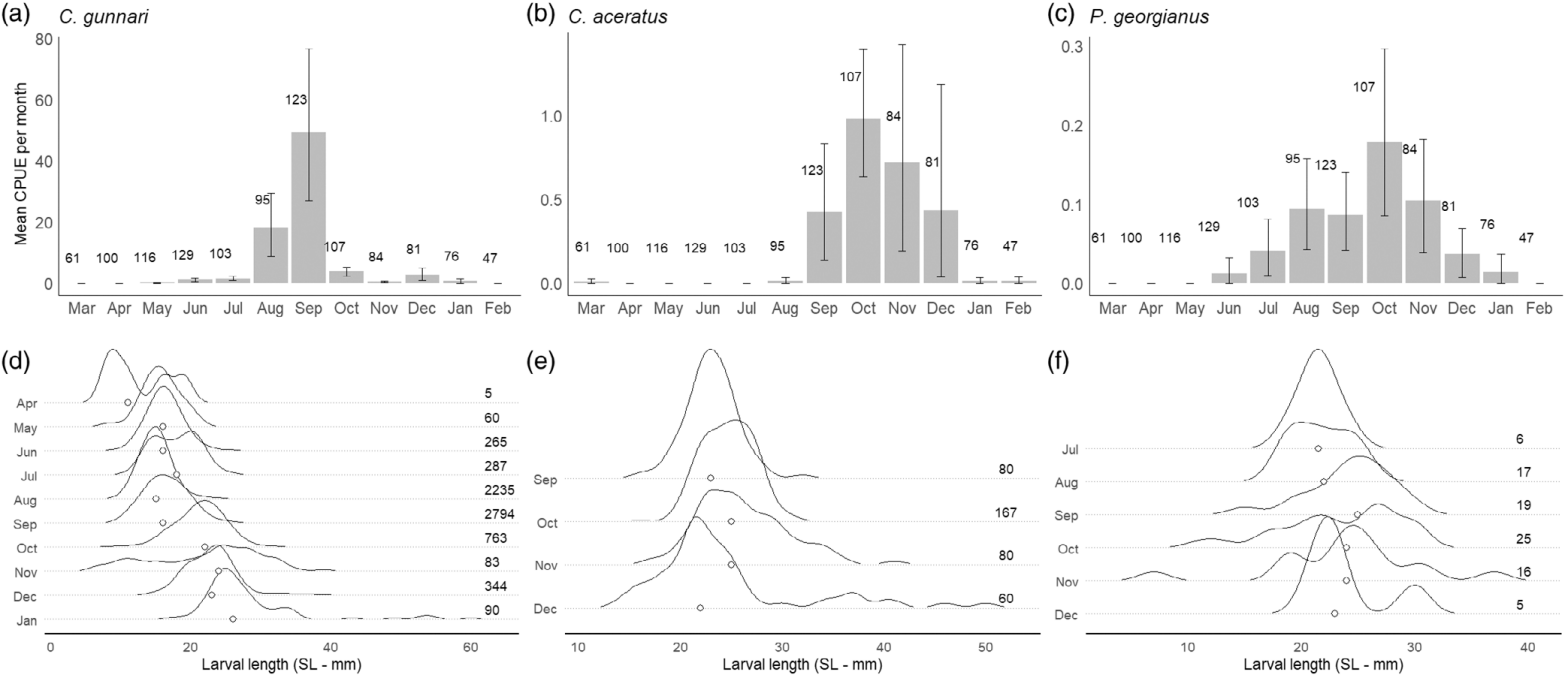
Mean monthly catch per unit effort (individuals caught per 1000 m^3^ swept) from plankton trawls conducted between 2001 and 2022 for *Champsocephalus gunnari* (a; *n* = 15,926 individuals caught), *Chaenocephalus aceratus* (b; *n* = 444) and *Pseudochaenichthys georgianus* (c; *n* = 98) (error bars represent the 95% bootstrap confidence interval). Trawls per month are listed above the bars (a–c). Plots d–f demonstrate the frequency distribution of the monthly larval length data; white dots indicate the median length (SL mm). Monthly sample sizes are presented to the right of each plot (months with <5 individuals measured were excluded).

A total of 1122 plankton trawls were used, of which 721 were conducted within Cumberland Bay, between 2001 and 2022 (Figures 1 and 2). Larvae of *C. gunnari* were present in all months of the year, with the lowest mean CPUE recorded in February and the highest recorded in September, with 68% of all larvae recorded in this month (Figures 2a and S1). Alongside the major peak in September, the mean CPUE for *C. gunnari* also had a minor peak in mid‐summer (Figure 2). A total of 91.3% of the *C. gunnari* larvae were captured in Cumberland Bay. For *C. aceratus* (Figure 2b), 98.6% of the larvae were recorded between September and December (Figures 2b and S2) with a peak in CPUE in October, representing 41.2% of the total catch. Ninety‐five percent of *C. aceratus* larvae were captured in Cumberland Bay. Relatively low numbers of *P. georgianus* larvae were observed with 81.6% recorded in Cumberland Bay (Figure 2c). The CPUE data demonstrated an extended larval pulse from June to January, with its peak in October representing 28.6% of the catch (Figures 2c and S3). Larvae of all icefish species were absent in the four trawls completed at Shag Rocks (Figure S4).

For *C. gunnari* (Figure 2d), larvae ranged from 6 to 60 mm (SL), and there was evidence of three separate cohorts across the 12‐month period. The first was identified from April (median SL: 11 mm) to July (median SL: 18 mm), the second between July and November (median SL: 24 mm) and the final less prominent cohort was also observed between November and January (median SL: 26 mm) (Figure 2d). The data suggest that the larvae for *C. aceratus* (SL range: 14–50 mm) likely represented a single cohort with protracted hatching across these months (Figure 2e). However, a slight decrease was noted in larval median length in December (22 mm) in comparison to November (25 mm). The median length of *P. georgianus* (SL range: 7–44 mm) increased in size from July (21.5 mm) to September (25 mm). However, larvae in October (24 mm) and December (23 mm) had a smaller median length compared to the previous month (Figure 2f).

### 3.2 Maturity, length at 50% spawning and seasonal variation in sex ratio

The total size ranges for each species, including individuals that were not sexed, were 4–60 cm TL (males: 11–60 cm, females: 10–60 cm) for *C. gunnari* at South Georgia; 10–50 cm (males: 15–44 cm, females: 12–49 cm) for *C. gunnari* at Shag Rocks; 7–76 cm (males: 13–67 cm, females: 13–73 cm) for *C. aceratus* and 5–59 cm (males: 8–56 cm, females: 14–59 cm) for *P. georgianus*. No ripe male *C. aceratus* were recorded (stage 4), but the data included all maturity stages for the other species. Due to data collection priorities, no maturity data were collected for *C. aceratus* or *P. georgianus* in surveys conducted between 2012 and 2019.

**FIGURE 3:**
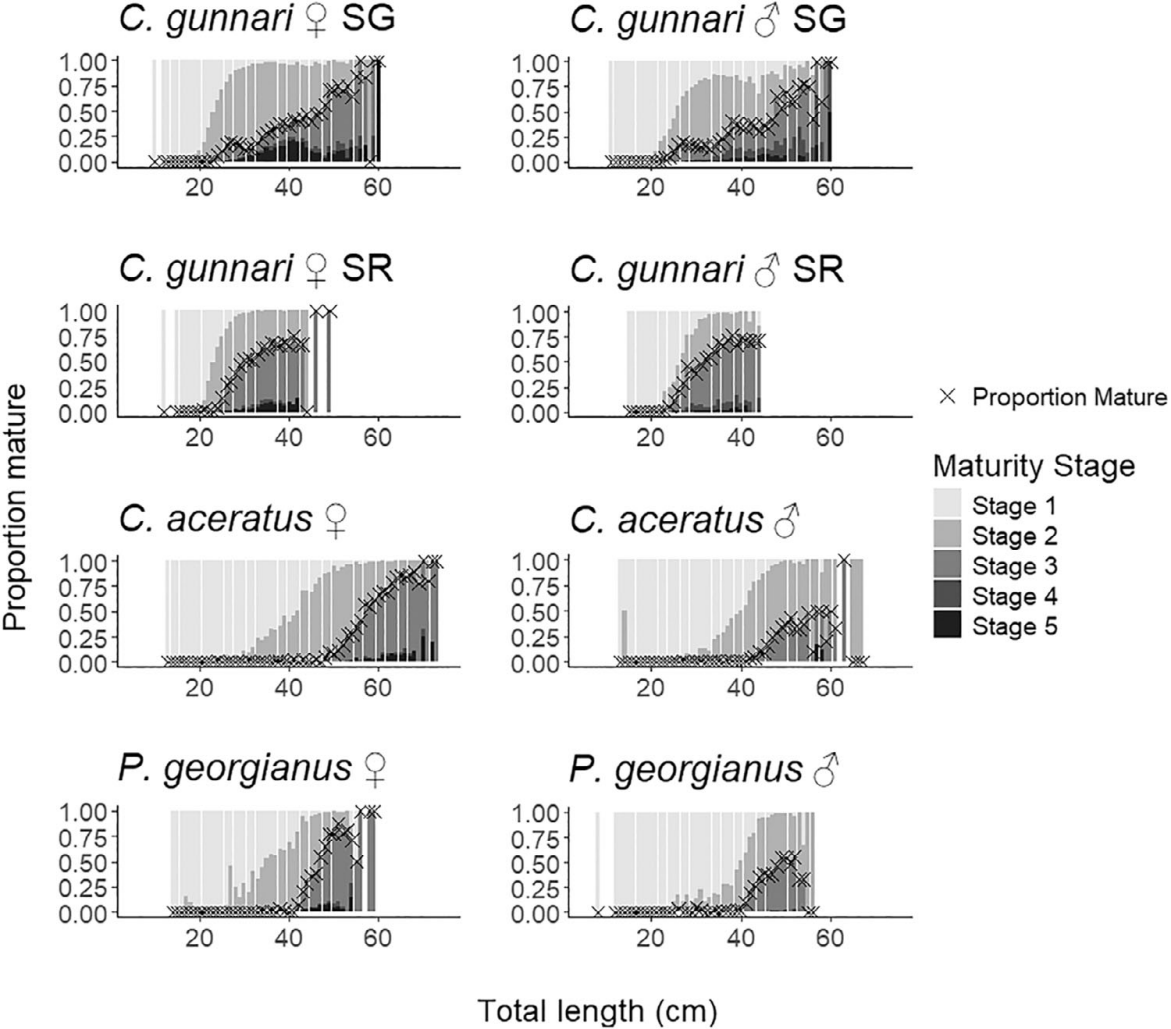
The proportion of mature (maturity Stage 3 and above) males (♂) and females (♀) for *Champsocephalus gunnari* (SG = South Georgia, *n* = 42,488), *C. gunnari* (SR = Shag Rocks, *n* = 10,171), *Chaenocephalus aceratus* (*n* = 13,922) and *Pseudochaenichthys georgianus* (*n* = 9051).

For *C. gunnari* around South Georgia, individuals first matured at 20 cm in both males and females (Figure 3). The proportion of mature individuals increased with size and plateaued several times before the populations achieved 100% maturity. For male *C. gunnari*, the proportion of mature individuals displayed plateau‐like transitions between 27–35 and 38–47 cm, either declining slightly or remaining approximately constant. Immature (stage 1) individuals were present up to 56 cm. Similar plateaus were observed for female *C. gunnari*, at 27–34, 40, 42 and 45 cm. At larger sizes, several reductions in the proportion of mature individuals were observed for both sexes; however, this may be due to low sample sizes. Albeit less distinct, a similar trend was seen among male *C. gunnari* sourced from Shag Rocks (Figure 3), where the proportion of mature individuals plateaued at 28–31, 35–37 cm, and peaked at 38 cm (0.77 proportion mature). No length class achieved 100% maturity, and low numbers of stage 1 male fish were observed up to 44 cm. Plateaus in maturity were noted in the females between 31–33, 36–39 and 41–46 cm before achieving 100% maturity. The smallest individuals recorded as mature were 23 cm for males and 21 cm for females. *C. gunnari* achieved a larger maximum size at South Georgia compared to Shag Rocks.

In male *P. georgianus*, the smallest mature individual was 26 cm (Figure 3). The proportion of mature individuals reached a maximum of 0.56 at 52 cm. Above this, the proportion of males at maturity Stage 3 or above decreased to zero at 55 and 56 cm. In contrast, male *C. aceratus* first attained maturity at 27 cm and achieved 100% maturity at 63 cm (*n* = 1). This was not maintained as larger individuals were categorised as maturity stage 2, suggesting that large mature males were not well represented in the data. No large virgin fish (stage 1) were observed for *C. aceratus* above 58 cm. Females of both species displayed a more consistent upward trajectory in the relationship between the proportion mature and total length. A slight reduction was observed within the larger length classes for female *C. aceratus* and *P. georgianus*. This is likely due to lower numbers of individuals caught within these size classes relative to smaller size classes within the dataset. Female *C. aceratus* first achieved maturity at 24 cm, whereas *P. georgianus* were 34 cm and larger than their male counterparts. Low numbers of virgin fish greater than 50 cm in length were observed in both sexes for *P. georgianus*. Females consistently displayed a larger L_50_ in comparison to males for each species, excluding the population of *C. gunnari* at Shag Rocks (Table 1). The bootstrap hypothesis test demonstrated that the L_50_ of males and females only significantly differed for *C. aceratus* (*p* < 0.01), with no difference in size for *C. gunnari* (South Georgia) (*p* = 0.36), *C. gunnari* (Shag Rocks) (*p* = 0.69) or *P. georgianus* (*p* = 0.95).

**TABLE 1:** The L_50_ values along with the 95% confidence intervals (calculated via the bootstrap method) for both male (♂) and female (♀) *Champsocephalus gunnari* (SG = South Georgia), *C. gunnari* (SR = Shag Rocks), *Chaenocephalus aceratus* and *Pseudochaenichthys georgianus*.

Species	Sex	L_50_ (cm)	Lower CI	Upper CI	*n*
*Champsocephalus gunnari* (SG)	***♂***	32.09	31.60	32.82	20,182
	*♀*	32.58	32.23	33.10	22,306
*C. gunnari* (SR)	***♂***	32.30	32.13	32.46	4994
	*♀*	32.25	32.11	32.39	5177
*Chaenocephalus aceratus*	***♂***	48.37	48.03	48.86	7343
	*♀*	59.12	59.04	59.20	6579
*Pseudochaenichthys georgianus*	***♂***	46.77	46.51	46.96	5062
	*♀*	48.00	47.77	48.17	3989

*Note*: The sample size (number of individuals who's maturity was graded per sex, per species) is also displayed.

**FIGURE 4:**
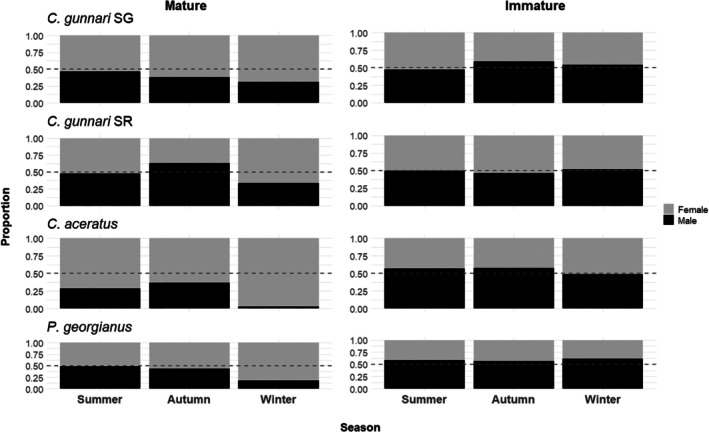
The sex ratio for mature and immature *Champsocephalus gunnari* (South Georgia, *n* = 42,488), *C. gunnari* (Shag Rocks, *n* = 10,171), *Chaenocephalus aceratus* (*n* = 13,922) and *Pseudochaenichthys georgianus* (*n* = 9051) for summer, autumn, and winter. The dashed grey line represents a 50% ratio.

For mature fish (Stage 3 and above), females were the dominant sex captured across all species and seasons, with the only exception being *C. gunnari* at Shag Rocks during autumn (Figure 4). Mature individuals of all species demonstrated the lowest proportion of males in the winter surveys, ranging from 33.3% of all individuals sampled for *C. gunnari* at Shag Rocks to a single male for *C. aceratus*. Similarly, only seven mature male *P. georgianus* were sampled in the winter surveys. For immature and resting individuals (stages 1 and 2), the dominance in the sex ratio was reversed where males were the most dominant sex for all species and survey seasons (Figure 4), except for *C. gunnari* (South Georgia) in the summer, *C. gunnari* (Shag Rocks) in the summer and autumn and *C. aceratus* in the winter.

### 3.3 Cohort identification and maturity

**FIGURE 5:**
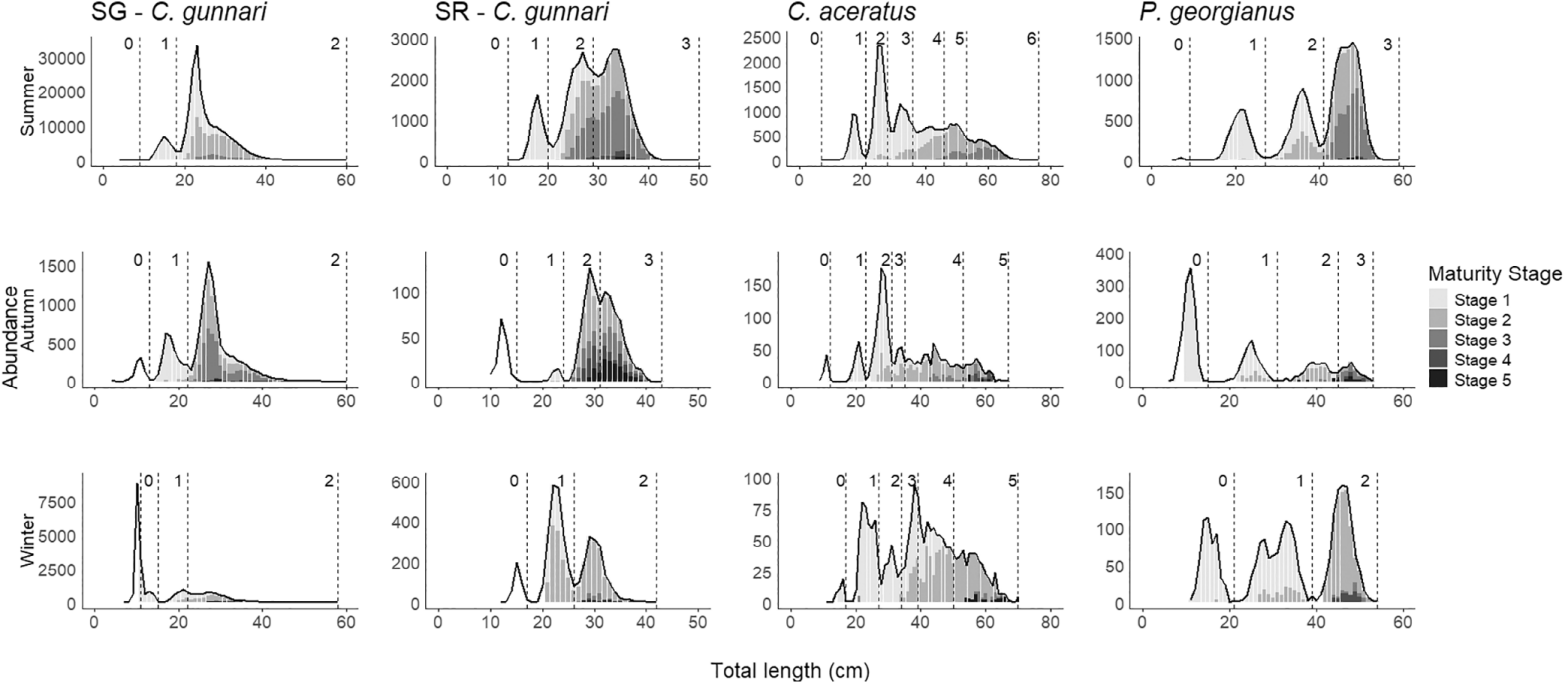
Length frequency analysis for *Champsocephalus gunnari* (South Georgia), *C. gunnari* (Shag Rocks), *Chaenocephalus aceratus* and *Pseudochaenichthys georgianus*, from surveys conducted between the Austral summer, autumn and winter. The dotted lines represent boundaries between cohorts that are likely to contain homogeneous age groups, guided by finite mixture models. Peaks within each cohort are numbered with their putative annual age. The shaded bars demonstrate the proportion of individuals sampled for both sexes that were categorised at each maturity stage for each centimetre. The sample size (*n*) for the length frequency analysis is detailed in Table S2.

For all species, a cohort with a putative age of 0+ was identified in the autumn and winter surveys but was predominantly absent during the summer surveys (Figure 5). Three length cohorts were identified for South Georgia's *C. gunnari*. Cohort 0+ were found in low numbers during the Austral summer surveys but were the most abundant cohort during the winter. The FMMs identified a secondary small peak for Cohort 0+ during the winter, between 12 and 15 cm. Cohort 1+ contained individuals predominantly at maturity stage 1, with a greater proportion of maturity stage 2 in the winter survey. Cohort 2+ likely included individuals of multiple ages. During the summer and winter surveys, the predominant maturity stage identified in this cohort was maturity stage 2. For all survey seasons, individuals classified as maturity stage 1 were identified in the largest size cohort (Cohort 2+). During the autumn surveys, individuals classified as mature increased in representation.

In comparison, three or four cohorts were identified for *C. gunnari* at Shag Rocks. Cohort 0+ was absent during the summer, but present during the autumn and winter surveys. What was likely to be a cohort representing 1‐year‐old fish including individuals at maturity stages 1 and 2 was present in all seasons. Two larger cohorts, comprising a mixture of immature and mature individuals, were identified, although the FMMs had difficulty identifying Cohort 2+ in the autumn. Cohort 3+ was absent during the winter survey. Individuals greater than 43 cm TL were absent in both the autumn and winter surveys.

For *C. aceratus*, six cohorts were identified for the summer surveys (Cohort 0+ was absent). Cohorts up to age class 2 were clearly visible, containing individuals from maturity stages 1 and 2. For Cohort 3+ and above, the FMMs were less able to identify clear cohorts, with autumn and winter exhibiting six cohorts. Cohorts 3+ and above included mature individuals from maturity stages 3, 4 and 5.

*P. georgianus* length frequency data demonstrated the most distinct putative age cohorts. Four cohorts were identified; however, Cohort 3+ was absent in the winter surveys, and low numbers of Cohort 0+ were present in the summer surveys. Cohorts 0+ and 1+ included individuals from maturity stages 1 and 2, whereas mature individuals associated with maturity Stage 3 and above began to appear in Cohort 2+. The final cohort contained the majority of the mature individuals.

## 4 DISCUSSION

Although there was some variation in the sampling strategy over the 37‐year survey period (Saunders et al., 2022), findings from the long‐term dataset reveal clear overarching trends, suggesting that each icefish species exhibits distinct life‐history strategies. This includes the indication that *C. gunnari* undergo multiple spawning pulses in a single year, whereas *C. aceratus* and *P. georgianus* complete a single reproductive event per annum as evidenced by the pulses in the CPUE of larvae. Length at spawning and length frequency analysis indicated that maturation varied between species, with *C. gunnari* potentially exhibiting two strategies where part of the population matures at a smaller size, whereas others mature at a larger size. In comparison, *C. aceratus* and *P. georgianus* invest in somatic growth before reaching maturity at a larger size. There was evidence of similarities in life‐history characteristics among species, which included females generally achieving maturity at a larger size in comparison to males as shown by the L_50_ analysis. Furthermore, assessments of the proportion of each sex per season demonstrated biased sex ratios, with mature males decreasing in number during the winter months for all species.

### 4.1 Larval pulses

The larval analysis suggests that *C. gunnari* spawns several times in a single year. A primary pulse was observed in the CPUE of *C. gunnari* larvae during mid to late winter, which was accompanied by a secondary smaller pulse in the summer (December and January). Multiple cohorts were observed in the larval length data, and similar patterns have been observed in previous research (Belchier & Lawson, 2013; North, 1998). This is consistent with life‐history traits observed on the Kerguelen shelf (Southern Indian Ocean), where *C. gunnari* is believed to exhibit spatial and temporal separation in their reproductive behaviour, with one population spawning in inshore canyons in the winter and the other across the slope of Skif Bank in the autumn (Duhamel et al., 2011). Possible explanations for the multiple pulses could be batch spawning, where females spawn multiple times throughout the season to hedge against unfavourable conditions and mitigate mortality in their offspring (Hočevar et al., 2021; Militelli & Macchi, 2004). A further possibility is that *C. gunnari* employs alternative reproductive tactics, with individuals exhibiting varying levels of energy expenditure in reproduction, resulting in differences in larval growth rates (Taborsky, 2008). Previous research has supported this hypothesis, demonstrating that the growth rates of larvae vary depending on which seasonal pulse they originate (Belchier & Lawson, 2013; North, 1998). However, it remains unconfirmed whether the variation in growth and abundance was caused by a change in reproductive tactics or due to seasonal environmental covariates, such as temperature and food availability (North, 1998). As sampling was uneven across time and space for this study, with a substantial proportion of the data sourced exclusively from Cumberland Bay, further research is required to confirm if *C. gunnari* consistently employs multiple spawning strategies and whether this is spatially restricted. More extensive plankton trawls could be conducted across Shag Rocks to assess whether larvae of *C. gunnari* are present in this region.

Larval catches of *C. aceratus* were primarily constrained between September and December with a single peak, and larval length demonstrated a continuous increase, excluding December. Previous research on the fecundity of *C. aceratus* supports this as it has been classified as a total spawner, with spermatogenesis and oogenesis appearing to be synchronous, resulting in a single annual spawning event (Riginella et al., 2016). *C. aceratus* has been confirmed to exhibit nesting and parental care as part of their reproductive strategy in other regions (Detrich et al., 2005). The high energy expenditure required for such a strategy supports the likelihood that this species will reproduce annually. Hatching times have been reported to range from July to December with a peak in November in the South Shetland Islands (Kock & Kellermann, 1991; Riginella et al., 2016). As the incubation period is expected to take less time at lower latitudes due to warmer conditions (Kock, 2005), the larval peak at South Georgia approximately coincides with when this would be expected to occur. The reduction in median length observed in December may be the result of ontogenetic habitat shift (Kock, 2005), where larger and more developed larvae transit to more demersal habitats, reducing their catchability.

Our observations suggest that *P. georgianus* employs a single extended larval pulse between June and January. Although a small secondary peak was observed in August and the median length did reduce in comparison to previous months, it is unclear whether this is representative of the species' ecology or due to the low sample size. There is currently very little information regarding the reproductive strategies of *P. georgianus*. Evidence suggests that, similar to *C. aceratus*, they focus their energy on somatic growth at an early stage and reach sexual maturity at a larger size, possibly undertaking their first spawn between ages 4 and 6 years (Chojnacki & Palczewski, 1981; Traczyk & Meyer‐Rochow, 2019). Delayed maturity along with their relatively low fecundity and comparatively large egg size once again suggests a high energy expenditure for reproduction (Vanella et al., 2005), which may involve parental care. Similar conclusions are supported in Clarke et al. (2008), and as its closest relative, Jonah's icefish *Neopagetopsis ionah* Nybelin 1947 (Near et al., 2003) has also been confirmed to provide parental care for its offspring (Purser et al., 2022). Studies on reproductive biology in the Scotia Arc have estimated that *P. georgianus* spawn in April (Vanella et al., 2005), and other research has estimated that hatching takes place towards the end of the winter (Chojnacki & Palczewski, 1981). The larval CPUE peak of October in this study is relatively consistent with these earlier estimates; nevertheless, caution should be applied when these results are interpreted due to the low number of larvae recorded.

Lower fecundity has been observed in both *C. aceratus* and *P. georgianus* in comparison to *C. gunnari* (Militelli et al., 2015). This may be further evidence that *C. aceratus* and *P. georgianus* consistently expend more resources on producing larger, more developed eggs and potentially undertake parental care. However, *P. georgianus* exhibited an extended larval phase, lasting 8 months (June to January), whereas *C. aceratus'* main pulse lasted 4 months (September to December). The extended larval recruitment period is potentially a bet‐hedging tactic utilised to avoid competition during development, as the diet of adult *P. georgianus*, which is largely based on krill, has overlaps with the diet of *C. gunnari* (Kock & Jones, 2005). This has been shown in other species, where spawning traits are engaged to cope with environmental uncertainty and to avoid direct competition (Nakayama et al., 2011). Alternatively, *P. georgianus* larvae may develop at a slower rate in comparison to the other two icefish species. Further specific research will be required to confirm whether the extended pulse is derived from a single cohort or due to an extended spawning period.

### 4.2 Putative age cohorts

For all three icefish species, individuals categorised within Cohort 0+ were generally absent during the summer surveys, as larvae would not have settled to a demersal habitat or reached a size vulnerable to the demersal trawl. Three size cohorts were consistently identified for *C. gunnari* around South Georgia; however, due to the multiple spawns in a given year these were less distinct and were likely to include fish of multiple age classes. Cohort 0+ fish were captured during the autumn, and in their greatest numbers during the winter. This is likely when the largest pulse of *C. gunnari* larvae from September became catchable by the demersal trawl survey. The existence of multiple spawning periods is supported further by the secondary peak in Cohort 0+ during the winter. The secondary pulse suggests that fish within this length range either hatched earlier or grew at a faster rate compared to the bulk of the cohort. The larval abundance and length frequency analysis support the hypothesis that sub‐sections of *C. gunnari* spawn at different periods and employ different life‐history strategies in the sense of developmental plasticity. However, sampling effort was uneven across seasons, which may impact the interpretation of the results.

Unlike at South Georgia, it was possible to divide Shag Rocks *C. gunnari* into four separate cohorts. Furthermore, the secondary peak observed in Cohort 0+ during the winter for *C. gunnari* at South Georgia was not identified at Shag Rocks. Alongside the differences in trends in maturity, this indicates that the life‐history strategies of *C. gunnari* at Shag Rocks are not consistent with those observed around South Georgia (Kuhn & Gaffney, 2006; North, 2005), and they may conduct a single discrete spawning period each year, resulting in more distinct cohorts.

The discrete annual spawning of *C. aceratus* made it possible to differentiate between distinct cohorts across multiple age classes, with the final cohort possibly representing individuals above 6 years of age. Previous research along the Southern Scotia Ridge, which used otoliths for ageing, estimated that C. *aceratus* has a maximum life span of 17 years with maturity first occurring at 14 years (Riginella et al., 2016). These findings suggest that individuals do not initially direct energy towards reproductive maturity and instead focus their energy on somatic growth. As observed during this study, mature individuals were recorded at low levels in lengths associated with Cohort 4+, suggesting that the species does not achieve sexual maturity until at least 4 years of age. The delayed sexual maturity compared to *C. gunnari* (Kock, 2005) may be due to the demands of nest guarding, particularly for males, where larger individuals are likely to achieve greater success (DeMartini, 1987). The finite mixture models had difficulty identifying distinct cohorts containing mature individuals, for example, those larger than Cohort 3+, (Reid et al., 2007). The increasing difficulty in identifying clear‐size cohorts suggests that this is the period where somatic growth begins to slow, and energy is directed towards attaining reproductive maturity.

*P. georgianus* had the most distinctive annual cohorts, with four detected in summer and autumn, and three in the winter. The distinctive nature of the peaks is a strong indication that the species reproduces once per annum. Mature individuals were generally confined to Cohort 2+ (winter) and 3+ (summer and autumn), and demonstrate the species' early investment in somatic growth and late sexual maturation in comparison to the earliest maturing *C. gunnari* at 20 cm (Kock, 2005). The lack of multiple cohorts with mature individuals suggests either a halt in somatic growth or could be a sign of semelparity within this species. Although this has not previously been suggested in *P. georgianus*, male *N. ionah* are generally in poor condition post‐spawn due to the high‐energy expenditure associated with reproduction and may frequently starve to death during the nest guarding phase (Biebow et al., 2014; La Mesa et al., 2021). Semelparity often evolves in species where the survival ratio of juveniles to mature individuals is high (Christiansen et al., 2008; Schaffer, 1974), which could be expected from a species which has a high energy expenditure into reproduction, such as nesting icefish.

### 4.3 Maturity and sex ratios

It appears that male and female *C. gunnari* at South Georgia potentially employ multiple life‐history strategies as some mature at a smaller size, achieving maturity as small as 20 cm TL, whereas other individuals remain virgins at over 50 cm TL (Everson et al., 1996). Variable reproductive phenotypes within a single species, such as size at maturity, are relatively common in male fish and provide information on the potential reproductive strategies they employ, such as alternative reproductive tactics (Mascolino et al., 2016). In other species, some individuals will delay their maturation and redirect energy expenditure towards somatic growth to increase fitness and develop into large territorial fish, which may take part in physically demanding tasks, such as parental care (Taborsky, 2008). This energy‐intensive strategy is commonly referred to as an equilibrium strategist (Winemiller & Rose, 1992). Other populations, often described as opportunistic strategists (McCann & Shuter, 1997), will invest more energy in reproductive growth to achieve sexual maturity at a smaller size. These individuals will not participate in reproductive activities which require a high energy expenditure but instead may attempt to undertake reproductive parasitism by covertly fertilising eggs that are being guarded by territorial males or will fertilise eggs outside of a nest and provide no parental care (Kvarnemo et al., 1995; Taborsky, 2008). Although there is uncertainty regarding the exact reproductive strategy that *C. gunnari* employs, with hypotheses ranging from high‐energy investments to pelagic broadcast spawning (Christiansen et al., 1998; Kock & Everson, 2003; Militelli et al., 2015), the result from this analysis suggests that multiple theories may be correct. Evidence suggests that *C. gunnari* employs alternative reproductive tactics, where large males have high‐cost reproductive strategies (nest building and parental care), and smaller individuals employ low‐cost reproductive strategies which may include acting as sneaker males (with zero parental care) as observed in other nest building species (Watanabe et al., 2008).

At Shag Rocks, male *C. gunnari* did not achieve 100% maturity at any size. The absence of large mature males could be due to a proportion of the population not spawning in any of the surveyed periods, or because they depart from Shag Rocks. Furthermore, plateaus in maturation were less prominent in both sexes, and the maximum length of fish was overall smaller in comparison to South Georgia (North, 2005). These traits may suggest that reproductive strategies vary spatially and a larger proportion of the population at Shag Rocks may favour maturation at a small size over investing in somatic growth to mature at a larger size. However, this could also indicate that large *C. gunnari* migrate from Shag Rocks to South Georgia after maturity is achieved.

In contrast, individuals from both sexes in *C. aceratus* and *P. georgianus* achieved maturity at a larger size, supporting the theory that they are purely equilibrium strategists (Riginella et al., 2016), and invest substantially in somatic growth before reproducing. It was noted that large mature males in *C. aceratus* and *P. georgianus* were captured in low numbers and did not consistently achieve 100% maturity even in the largest length classes, suggesting that the mature male life stage was poorly sampled. This is also reflected in the results from the sex ratios over each season, which were highly biased towards mature females during the winter in all species. This biased sex ratio may be explained by males and females exhibiting differential parental duties, as shown in multiple other fish species, including *C. aceratus* (Ferrando et al., 2014; Goldberg et al., 2020; Hourigan & Radtke, 1989; Jones & Near, 2012; Kock et al., 2008; Rae & Calvo, 1995). During the winter months, it is possible that male *C. aceratus* were undertaking nest‐guarding duties in areas that were poorly sampled, such as rocky substrata or the glacial troughs radiating from the fjords across South Georgia's shelf. This is also supported by the lack of ripe males sampled throughout the history of the demersal survey.

With regard to *P. georgianus*, only seven mature males were captured during the winter, which suggests a similar trend to *C. aceratus* where the behaviour of mature males reduces their catchability during the winter, possibly due to nest guarding. Alternatively, the skewed ratio may instead reflect increased male mortality resulting from the energetic costs of reproduction. Heightened male mortality associated with reproduction has been observed in other species, frequently due to male–male competition or reduced fitness associated with the burden of providing parental care (DeMartini, 1987; Forsgren et al., 2004; Lindström, 2001; Marconato et al., 1993; Rios‐Cardenas & Webster, 2005). Furthermore, in some species males are semelparous, whereas females exhibit iteroparity (Christiansen et al., 2008). The continued low abundance of mature male *C. aceratus* in summer is indicative of a long and costly parental care strategy, with an incubation period estimated to last around 120 days, which may include some level of fasting (Kock & Kellermann, 1991). As large mature females were comparatively well sampled, it suggests that their behaviour and subsequent catchability or their actual abundance is less impacted by season, supporting the hypothesis that they have a low investment in parental care. However, further research will be required to determine the true cause of the skewed sex ratio in mature fish.

*C. aceratus* was the only species where the L_50_ for females was significantly larger than the L_50_ for males. The large size attained by females before achieving maturity is likely due to the energetic cost of producing eggs, which are large in comparison to other icefish species (Militelli et al., 2015) and is further evidence of the large energy expenditure in the reproductive strategies of *C. aceratus*. It should be noted that the L_50_ values calculated in this study included data collected outside of each species' spawning season, which may impact the robustness of the values. Similarly, although the five‐point maturity scale developed by Kock and Kellermann (1991) is routinely used for multiple Southern Ocean fish species (Militelli et al., 2015; Novillo et al., 2021), its categories can limit biological interpretation. For example, maturity stage 2 can include individuals that have previously spawned and those that are preparing to spawn for the first time. These mixed stages can mask shifts in a species life history and impact maturity analysis. Therefore, further efforts should be made to improve the resolution of the five‐point scale.

## 5 CONCLUSION

The findings from this study suggest that South Georgia and Shag Rocks' icefish exhibit diverse life‐history strategies. Notably, it appears that *C. gunnari* may exhibit alternative reproductive tactics. In contrast, both *C. aceratus* and *P. georgianus* are more likely to adopt an equilibrium reproductive strategy, where they exclusively invest in somatic growth and mature at a large size, undertaking spawning once per annum. The late maturation, single larval pulse and biased sex ratios during the winter surveys suggest a consistent high investment in reproduction in these two species, potentially including parental care. Due to the inherent limitations of inferring some life‐history traits without direct observations, future efforts should aim to confirm the method, timing and location of reproductive strategies within each species. This information is vital for continued sustainable management in the region.

## AUTHOR CONTRIBUTIONS

Huw W. James, Fabrice Stephenson, Philip R. Hollyman, William D. K. Reid and Martin A. Collins conceptualised the study. Philip R. Hollyman, William D. K. Reid and Martin A. Collins participated in the data collection and fieldwork. Huw W. James conducted the analyses and wrote the manuscript. Huw W. James and Timothy Jones developed the methodology for some of the analyses. Timothy Jones, Fabrice Stephenson, Philip R. Hollyman, William D. K. Reid and Martin A. Collins edited the manuscript.

## FUNDING INFORMATION

Natural Environment Research Council (NERC) and UK Research and Innovation (UKRI) via the IAPETUS2 Doctoral Training Partnership PhD studentship‐Grant number NE/S007431/1.

## Supporting information


**Figure S1.** Monthly mean catch per unit effort for *Champsocephalus gunnari* larvae from 2001 to 2022 alongside effort per month. Error bars represent the 95% bootstrap confidence intervals (truncated at zero).
**Figure S2.** Monthly mean catch per unit effort for *Champsocephalus aceratus* larvae from 2001 to 2022 alongside effort per month. Error bars represent the 95% bootstrap confidence intervals (truncated at zero).
**Figure S3.** Monthly mean catch per unit effort for *Pseudochaenichthys georgianus* larvae from 2001 to 2022 alongside effort per month. Error bars represent the 95% bootstrap confidence intervals (truncated at zero).
**Figure S4.** Locations of plankton surveys between 2001 and 2022 (midpoint of trawls – top map) (*n* = 1122) alongside the start points of trawls utilising a demersal trawl method conducted between 1986 and 2023 across the South Georgia and Shag Rocks continental shelves (bottom map) (*n* = 1776). Bathymetry data were curtailed at 1000 m (Hogg et al., 2016, 2017). The coastline data were sourced from South Georgia GIS (2019). The figure was produced using QGIS 3.34.5‐Prizren (QGIS Development Team, 2024).
**Table S1.** The survey details, including dates, gear configurations and vessels for all events during the demersal surveys conducted between 1986 and 2023.
**Table S2.** The sample size for the length frequency analysis of *Champsocephalus gunnari* (SG = South Georgia, SR = Shag Rocks), *Chaenocephalus aceratus* and *Pseudochaenichthys georgianus* across each survey season. Individuals were included where maturity was recorded but sex was not.
