# Supplementary material for: Contrasting life‐history strategies of three sympatric icefish species in the northern Scotia Sea
Source: J Fish Biol. 2026 Feb 10;108(6):1838–51. doi: 10.1111/jfb.70344 (PMC13357364; doi:10.1111/jfb.70344)
Supplement: Supplementary file 1 — Figure S1. Monthly mean catch per unit effort for Champsocephalus gunnari larvae from 2001 to 2022 alongside effort per month. Error bars represent the 95% bootstrap confidence intervals (truncated at zero). Figure S2. Monthly mean catch per unit effort for Champsocephalus aceratus larvae from 2001 to 2022 alongside effort per month. Error bars represent the 95% bootstrap confidence intervals (truncated at zero). Figure S3. Monthly mean catch per unit effort for Pseudochaenichthys georgianus larvae from 2001 to 2022 alongside effort per month. Error bars represent the 95% bootstrap confidence intervals (truncated at zero). Figure S4. Locations of plankton surveys between 2001 and 2022 (midpoint of trawls – top map) (n = 1122) alongside the start points of trawls utilising a demersal trawl method conducted between 1986 and 2023 across the South Georgia and Shag Rocks continental shelves (bottom map) (n = 1776). Bathymetry data were curtailed at 1000 m (Hogg et al., 2016, 2017). The coastline data were sourced from South Georgia GIS (2019). The figure was produced using QGIS 3.34.5‐Prizren (QGIS Development Team, 2024). Table S1. The survey details, including dates, gear configurations and vessels for all events during the demersal surveys conducted between 1986 and 2023. Table S2. The sample size for the length frequency analysis of Champsocephalus gunnari (SG = South Georgia, SR = Shag Rocks), Chaenocephalus aceratus and Pseudochaenichthys georgianus across each survey season. Individuals were included where maturity was recorded but sex was not. [file JFB-108-1838-s001.docx]

# Supplemental materials


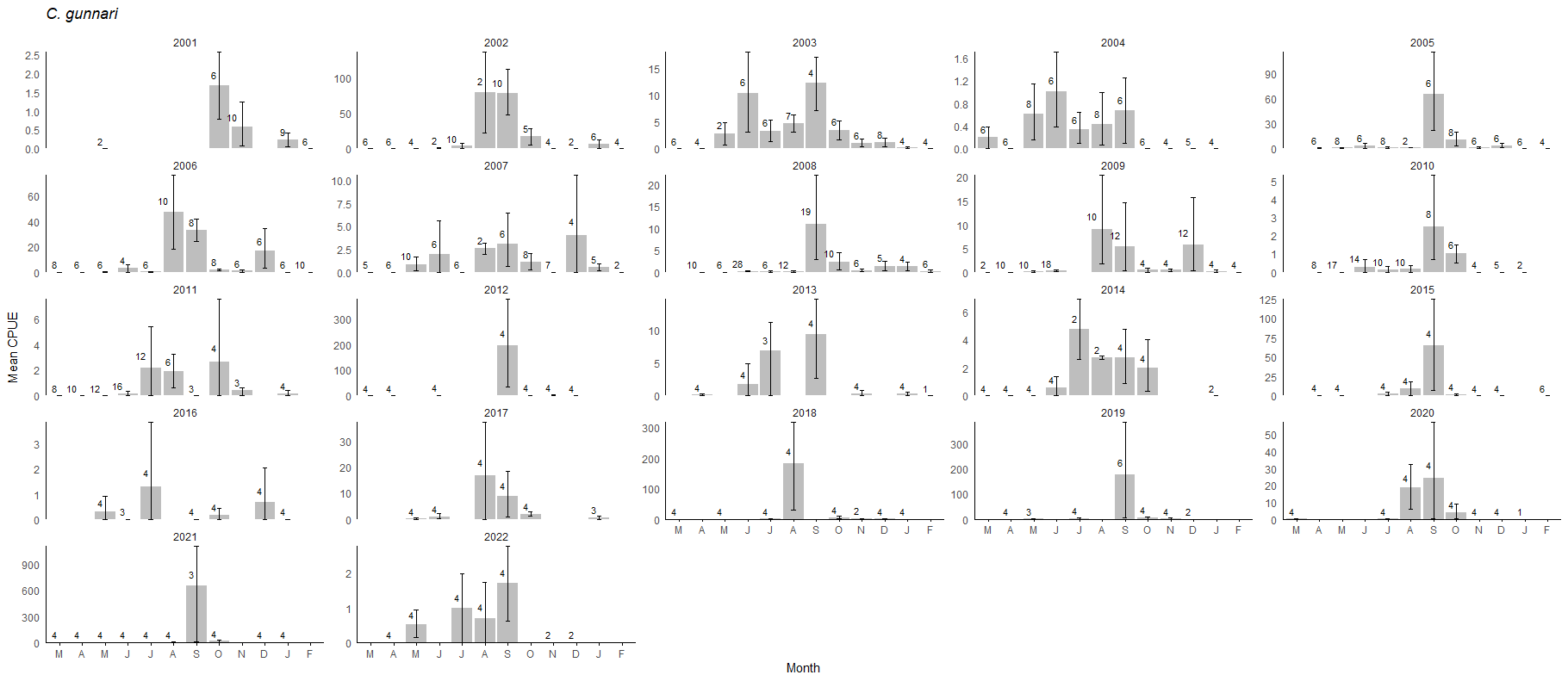


FIGURE S 1: Monthly mean catch per unit effort for *C. gunnari* larvae from 2001-2022 alongside effort per month. Error bars represent the upper (95%) and lower (5%) bootstrap confidence intervals (truncated at zero).


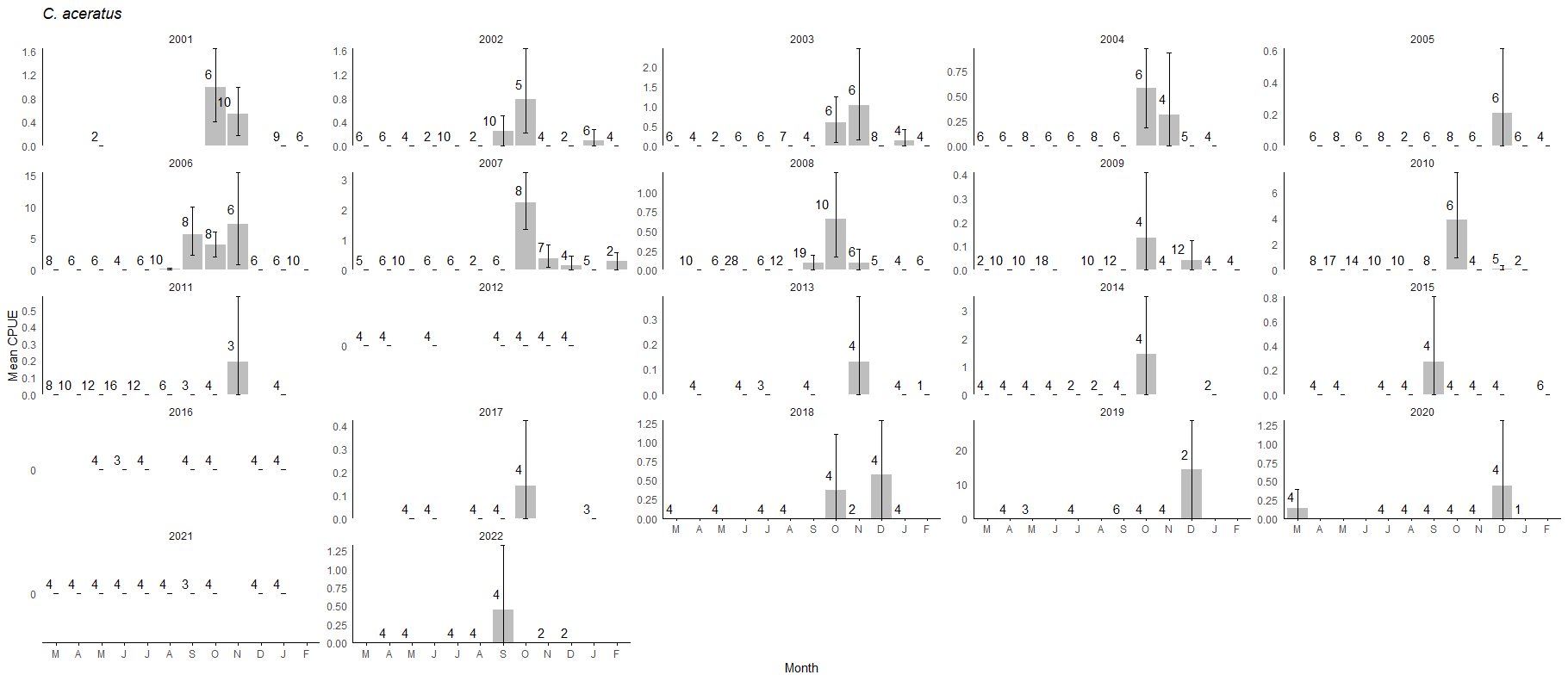


FIGURE S 2: Monthly mean catch per unit effort for *C. aceratus* larvae from 2001-2022 alongside effort per month. Error bars represent the upper (95%) and lower (5%) bootstrap confidence intervals (truncated at zero).


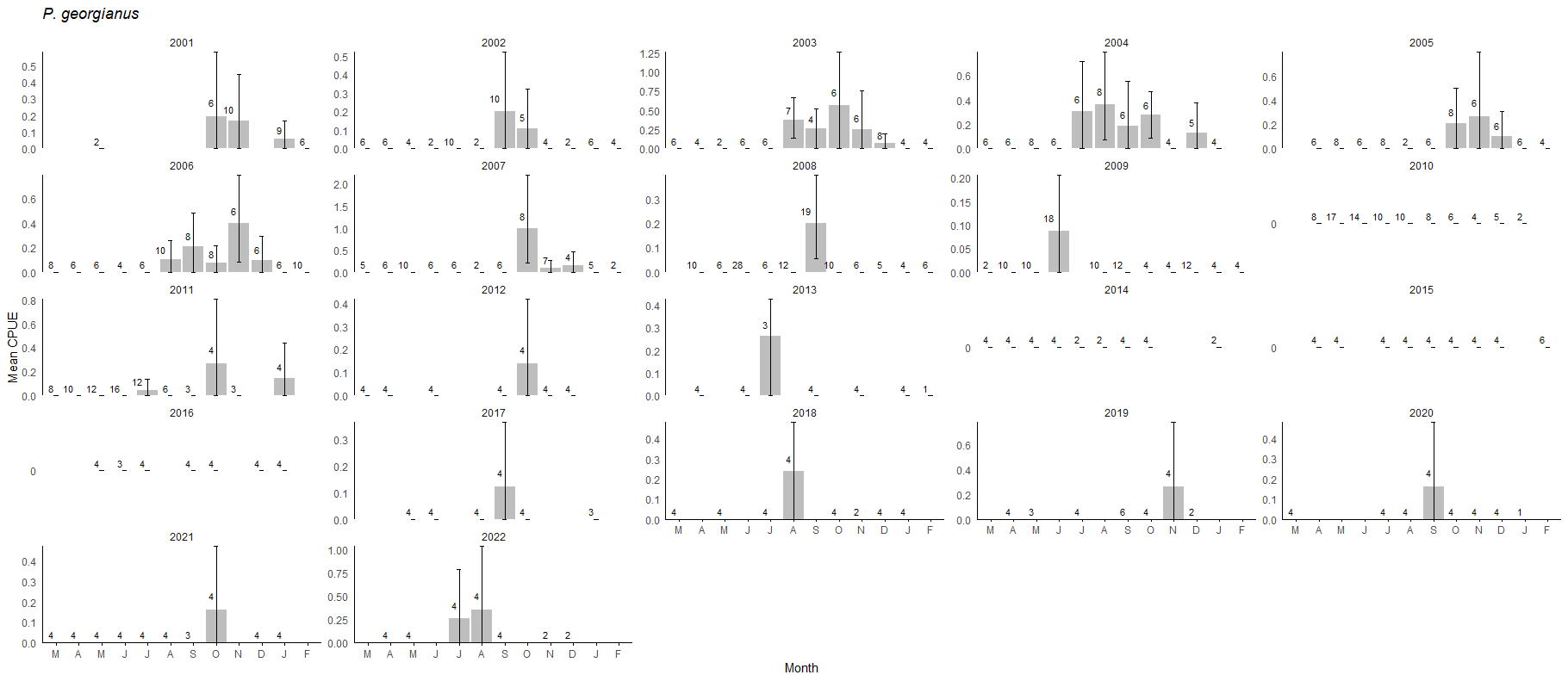


FIGURE S 3: Monthly mean catch per unit effort for *P. georgianus* larvae from 2001-2022 alongside effort per month. Error bars represent the upper (95%) and lower (5%) bootstrap confidence intervals (truncated at zero).


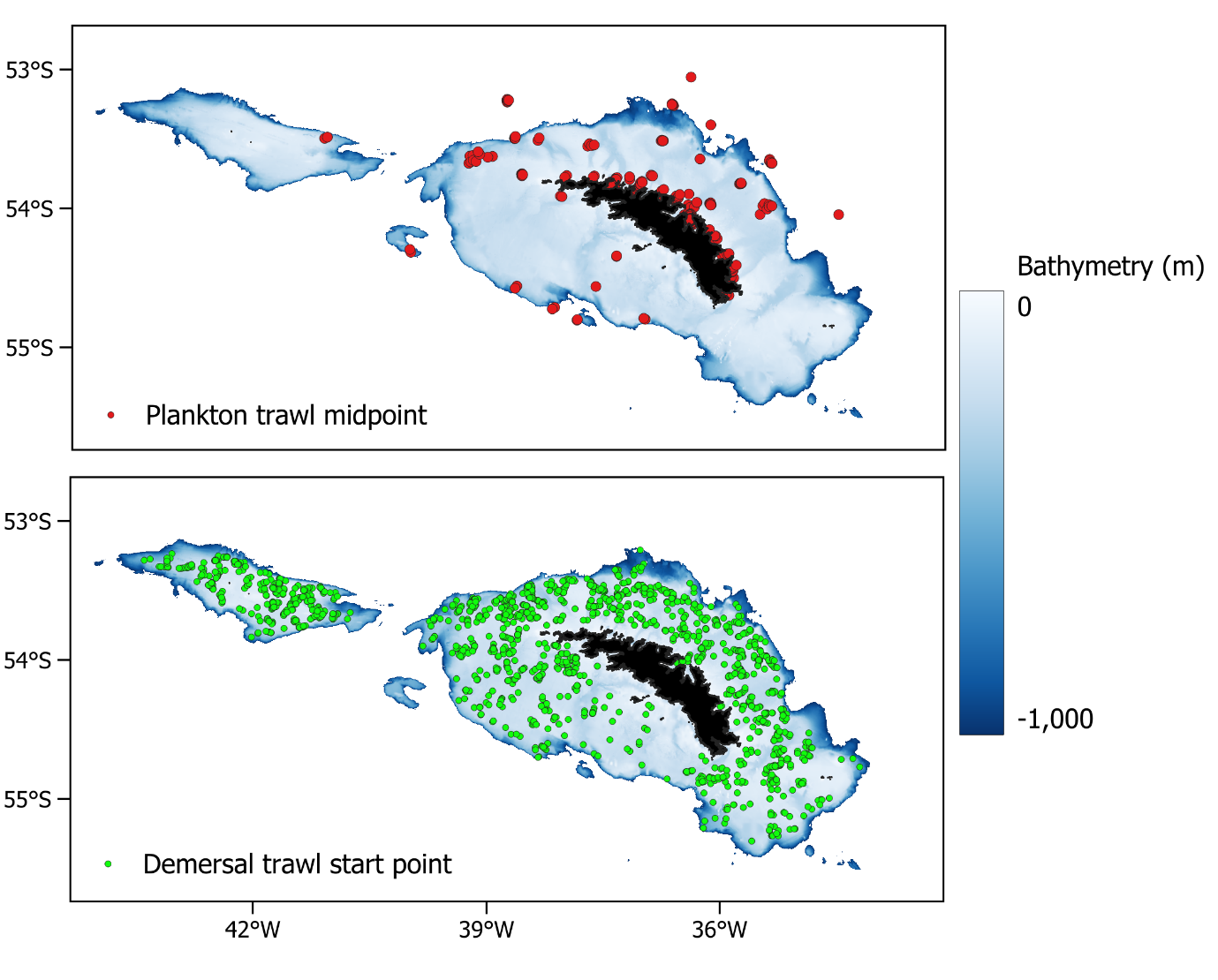


Figure S 4: Locations of plankton surveys between 2001-2022 (midpoint of trawls – top map) (*n*=1122) alongside the start points of trawls utilising a demersal trawl method conducted between 1986-2023 across the South Georgia and Shag Rocks continental shelves (bottom map) (*n*=1776). Bathymetry data was curtailed at 1000 m (Hogg et al., 2016, 2017). The coastline data was sourced from South Georgia GIS (2019). The figure was produced using QGIS 3.34.5-Prizren QGIS Development Team (2024).

TABLE S 1: The survey details, including dates, gear configurations, and vessels for all events during the demersal surveys conducted between 1986 and 2023.

| **Survey code** | **Start date** | **End date** | **Vessel** | **Net type** | **Net mesh size (mm)** | **Codend mesh size (mm)** | **South Georgia (*n*)** | **Shag Rocks (*n*)** |
| --- | --- | --- | --- | --- | --- | --- | --- | --- |
| SG87 | 29-Nov-86 | 17-Dec-86 | Profesor Siedlecki | B-454 OT | 80 | 20 | 93 | 11 |
| SG88 | 19-Dec-87 | 12-Jan-88 | Profesor Siedlecki | P32/36OT | 80 | 45 | 108 | 4 |
| SG89 | 01-Feb-89 | 14-Feb-89 | Profesor Siedlecki | P32/36OT | 80 | 40 | 55 | 0 |
| SG90 | 06-Jan-90 | 26-Jan-90 | Hill Cove | HC120 OT | 85 | 40 | 59 | 9 |
| SG91 | 22-Jan-91 | 11-Feb-91 | Falklands Protector | FP120 | 80 | 40 | 65 | 12 |
| SG92 | 03-Jan-92 | 26-Jan-92 | Falklands Protector | FP120 | 80 | 40 | 68 | 13 |
| SG94 | 04-Jan-94 | 08-Feb-94 | Cordella | FP120 | 80 | 40 | 68 | 13 |
| SG97 | 02-Sep-97 | 29-Sep-97 | Argos Galicia | FP120 | 80 | 40 | 43 | 12 |
| SG00 | 16-Jan-00 | 30-Jan-00 | Argos Galicia | FP120 | 80 | 40 | 30 | 11 |
| SG02 | 12-Jan-02 | 01-Feb-02 | FPV Dorada | FP120 | 80 | 40 | 44 | 19 |
| SG03 | 07-Jan-03 | 31-Jan-03 | FPV Dorada | FP120 | 80 | 40 | 28 | 10 |
| SG04 | 07-Jan-04 | 05-Feb-04 | FPV Dorada | FP120 | 80 | 40 | 44 | 21 |
| SG05 | 07-Jan-05 | 25-Jan-05 | FPV Dorada | FP120 | 80 | 40 | 28 | 14 |
| SG06 | 03-Jan-06 | 01-Feb-06 | FPV Dorada | FP120 | 80 | 40 | 47 | 19 |
| SG07 | 27-Aug-07 | 21-Sep-07 | FPV Dorada | FP120 | 80 | 40 | 34 | 15 |
| SG08 | 16-Apr-08 | 30-Apr-08 | Sil | FP120 | 80 | 40 | 51 | 19 |
| SG09 | 15-Jan-09 | 23-Jan-09 | Sil | FP120 | 80 | 40 | 54 | 19 |
| SG10 | 15-Jan-10 | 24-Jan-10 | Sil | FP120 | 80 | 40 | 59 | 16 |
| DW10 | 29-Jan-10 | 31-Jan-10 | Sil | FP120 | 80 | 40 | 6 | 0 |
| SG11 | 26-Jan-11 | 06-Feb-11 | Sil | FP120 | 80 | 40 | 71 | 16 |
| SG12 | 26-Jan-12 | 29-Jan-12 | New Polar | FP120 | 80 | 40 | 3 | 19 |
| SG13 | 22-Jan-13 | 29-Jan-13 | Sil | FP120 | 80 | 40 | 54 | 16 |
| SG15 | 13-Jan-15 | 23-Jan-15 | New Polar | FP120 | 80 | 40 | 54 | 23 |
| SG17 | 30-Jan-17 | 07-Feb-17 | Sil | FP120 | 80 | 40 | 53 | 19 |
| SG19 | 27-Jan-19 | 05-Feb-19 | Sil | FP120 | 80 | 40 | 56 | 17 |
| DW19 | 05-Feb-19 | 05-Feb-19 | Sil | FP120 | 80 | 40 | 3 | 0 |
| SG21 | 08-May-21 | 28-May-21 | Robin M Lee | FP120 | 80 | 40 | 57 | 19 |
| SG23 | 01-Feb-23 | 10-Feb-23 | Robin M Lee | FP120 | 80 | 40 | 56 | 19 |

TABLE S 2: The sample size for the length frequency analysis of *Champsocephalus gunnari* (SG = South Georgia, SR = Shag Rocks), *Chaenocephalus aceratus,* and *Pseudochaenichthys georgianus* across each survey season. Individuals were included where maturity was recorded but sex was not.

| Season | Length frequency/Maturity stage | *Champsocephalus gunnari* (SG) | *Champsocephalus gunnari* (SR) | *Chaenocephalus aceratus* | *Pseudochaenichthys georgianus* |
| --- | --- | --- | --- | --- | --- |
| Summer | Length-frequency | 231,823 | 38,590 | 34,657 | 20,168 |
|  | Maturity stage 1 | 14,677 | 1720 | 7025 | 3178 |
|  | Maturity stage 2 | 19,867 | 3235 | 4378 | 2855 |
|  | Maturity stage 3 | 4043 | 3858 | 1652 | 2061 |
|  | Maturity stage 4 | 588 | 277 | 13 | 27 |
|  | Maturity stage 5 | 926 | 160 | 76 | 90 |
| Autumn | Length-frequency | 12,813 | 1100 | 1862 | 2425 |
|  | Maturity stage 1 | 489 | 49 | 166 | 79 |
|  | Maturity stage 2 | 743 | 166 | 226 | 94 |
|  | Maturity stage 3 | 766 | 85 | 41 | 38 |
|  | Maturity stage 4 | 12 | 62 | 14 | 12 |
|  | Maturity stage 5 | 38 | 61 | 5 | 12 |
| Winter | Length-frequency | 25,083 | 4110 | 2011 | 2395 |
|  | Maturity stage 1 | 1031 | 95 | 289 | 397 |
|  | Maturity stage 2 | 1180 | 400 | 332 | 353 |
|  | Maturity stage 3 | 28 | 22 | 14 | 14 |
|  | Maturity stage 4 | 4 | 3 | 1 | 0 |
|  | Maturity stage 5 | 254 | 14 | 19 | 26 |

**References**

Hogg, O. T., Huevenne, V., Griffiths, H., Dorschel, B., & Linse, K. (2017). *A Bathymetric Compilation of South Georgia, 1985-2015.* [Data set]. Polar Data Centre; British Antarctic Survey, Natural Environment Research Council; Cambridge, CB3 0ET, UK. https://doi.org/10.5285/CE8BF6BE-4B5F-454C-9165-73AB4C3BAF23

Hogg, O. T., Huvenne, V. A. I., Griffiths, H. J., Dorschel, B., & Linse, K. (2016). Landscape mapping at sub-Antarctic South Georgia provides a protocol for underpinning large-scale marine protected areas. *Scientific Reports*, *6*(1), 33163. https://doi.org/10.1038/srep33163

QGIS Development Team. (2024). *QGIS Geographic Information System (Version 3.34.5-Prizren). Open Source Geospatial Foundation.* https://qgis.org

South Georgia GIS. (2019). *Terrestrial: Coastline* [Data set]. https://sggis.gov.gs/data/physical_layers/terrestrial/shp/
